# Royal College of Surgeons Guideline Adherence on Improvement of Operative Notes: A Six-Month Closed Loop Audit

**DOI:** 10.7759/cureus.7970

**Published:** 2020-05-05

**Authors:** Dujanah S Bhatti, Raheel Ahmad, Ayesha Asad

**Affiliations:** 1 Surgery, Rawalpindi Medical College, Rawalpindi, PAK; 2 Surgery, Holy Family Hospital, Rawalpindi, PAK; 3 Anatomy, Quetta Institute of Medical Sciences, Quetta, PAK; 4 Anatomy, National University of Medical Sciences, Rawalpindi, PAK

**Keywords:** othopedics, operative notes, documentsation, rcs guidlines

## Abstract

Introduction

Every surgical procedure is followed by thorough and descriptive documentation. The Royal College of Surgeons (RCS) introduced official guidelines in 2014 on the proper documentation of operative notes. These guidelines are concise, targeted, and easy to adapt to any surgical specialty.

Methods and results

An audit was conducted from April to September 2019 at District Headquarter Hospital in Rawalpindi, Pakistan. We analyzed 215 operative notes of elective and emergency cases during the first three months (pre-implementation), and after comparing the notes against the RCS orthopedic notes guidelines, we developed a dedicated notes format for use in the second three months (post-implementation). A panel reviewed 235 notes postimplementation for legibility and compliance with the 2014 RCS guideline data.

Operative diagnosis was written in 80% pre-implementation notes and 100% of post-implementation notes. Pre-implementation of RCS guidelines, 78% of operative notes included the names of the anesthetist and the operative staff, 88% included assistant names, and 90% included the name of the operating surgeon. Post-implementation, these numbers increased to 92%, 93%, and 99%, respectively. All domains of the guideline were filled, and data point inclusion statistically significantly improved (0.001 < P < 0.005) after the implementation of RCS guidelines.

Conclusion

Following the RCS guidelines resulted in a significant improvement in all deficient fields in operative notes.

## Introduction

Operative notes are extremely important in all surgical specialties. They communicate details of the operation that otherwise would be impossible to decipher, and they play a crucial role in the research, audit, medico-legal importance, and overall performance of the surgical department. Unfortunately, these notes are often neglected and not given the necessary attention, time, or skills. This, in turn, accounts for most medical litigations. This trend has increased over the past two decades and is, unfortunately, increasing at an alarming rate [1]. To improve the quality of operative notes, the Royal College of Surgeons (RCS) introduced official guidelines in 2014 [2]. These guidelines are concise, targeted, and easy to adapt to any surgical specialty. They are considered the gold standard for operative notes and are the backbone for good medical practice, and their implementation has shown to dramatically increase the overall efficacy of operative notes [3].

The majority of hospitals in Pakistan have self-developed an operative notes format for operating rooms. Although they vary considerably, they are deficient in multiple areas when compared to the RCS guidelines. Our study aimed to identify the flaws and drawbacks in the operative notes format at a tertiary care hospital, as well as develop a new format for the operative notes, implement them into practice, and complete the audit cycle. District Headquarter Hospital is the primary referral unit for orthopedic cases, both elective and emergency. Unfortunately, no such audit could be found in the region. A simple template format was constructed; however, few studies have developed a computer-generated form [4].

## Materials and methods

The audit was conducted over six months from April to September 2019 at District Headquarter Hospital, which was the authors' training tertiary care hospital, located in the heart of Rawalpindi, Pakistan. The hospital has a well-established orthopedic department with elective and emergency centers and separate operative allocations.

**Figure 1:**
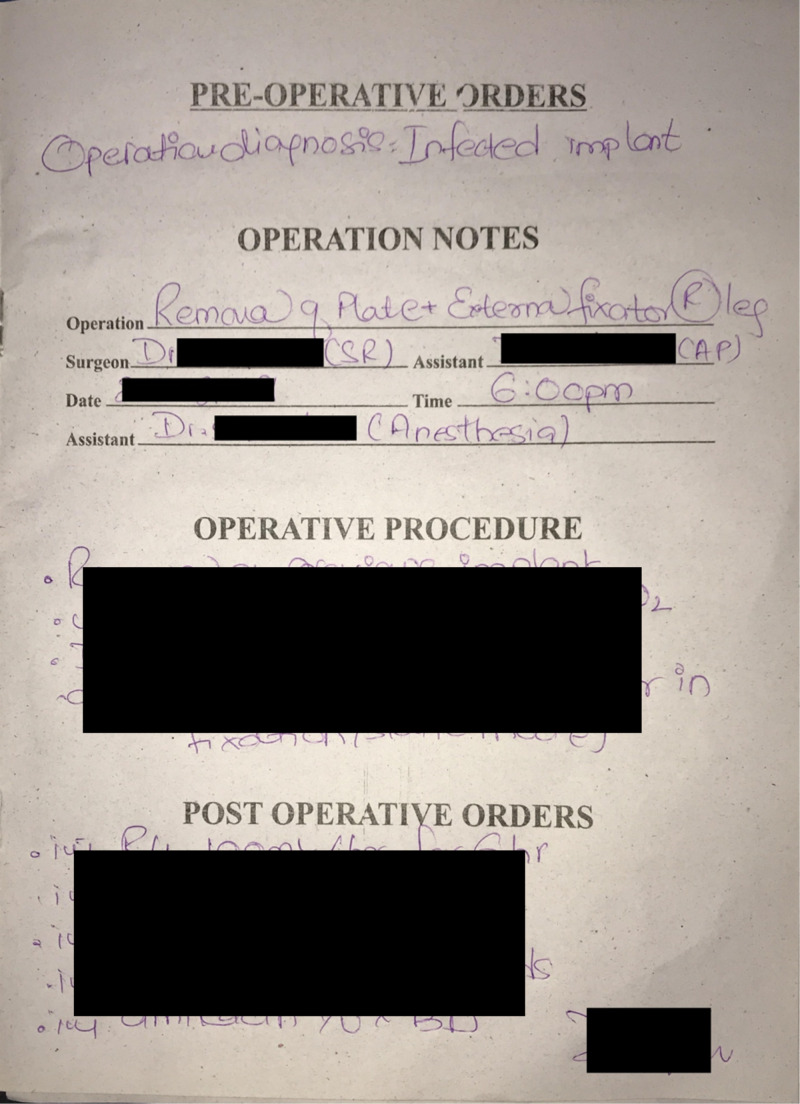
Operative notes prior to the implementation of the Royal College of Surgeons guidelines

The first three months were dedicated to analyzing the operative notes of elective and emergency cases. The notes were thoroughly inspected on all parameters and compared to the RCS orthopedic notes guidelines, and a dedicated orthopedic operation notes format was developed. The new format (Figure 1) was implemented in July 2019.

**Figure 2:**
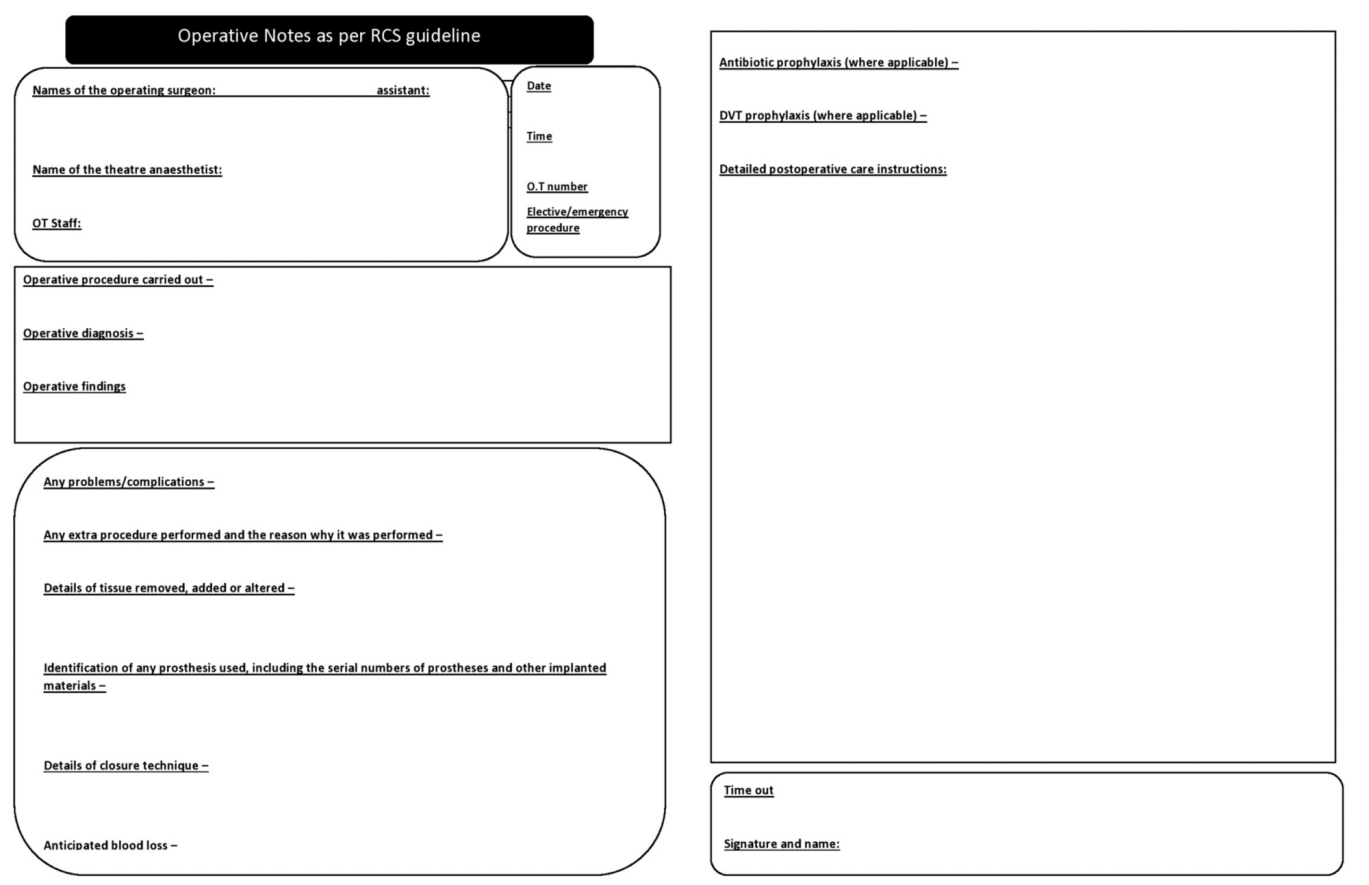
Improved operative notes after implementation of the Royal College of Surgeons guidelines

Orthopedic department staff and doctors of all grades were briefed about the use of the newly developed operative notes format. These guidelines were implemented with the help of poster presentations, short video presentations, and notices (Figure 2).

From July to September 2019, data were collected. A panel was convened, including two operating room staff, one consultant, and two postgraduate trainees, to review the legibility of the notes. Further analysis based on RCS guideline data on Good Surgical Practice (2014) was conducted using Microsoft Excel [2]. Chi-square test was used to measure the association between variables.

## Results

**Figure 3:**
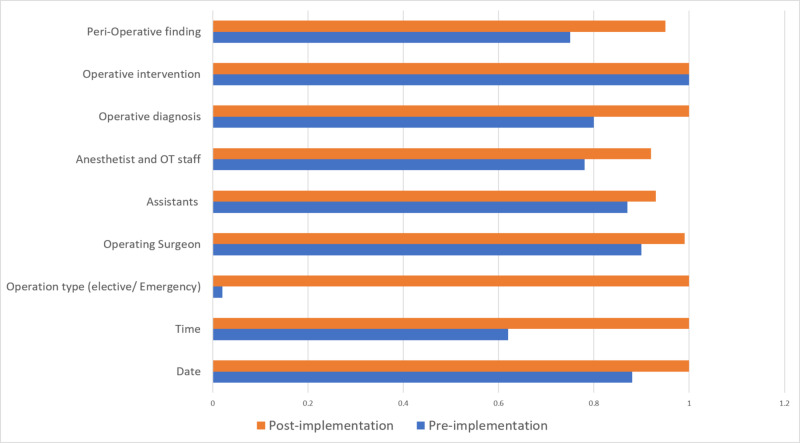
Operative notes after implementation of the Royal College of Surgeons guidelines OT, operation theater

A total of 500 operative notes were compared; 215 notes were studied before the implementation of RCS guidelines, and 235 were studied after implementation of RCS guidelines. Before the implementation of RCS guidelines, 72% of operative notes consisted of peri-operation findings (included in operative diagnosis or sub-heading in procedure notes), whereas, after the implementation of RCS guidelines, 96% of operative notes had peri-operation findings. Operative interventions were written in all operative notes pre- and post-implementation of RCS guidelines. Operative diagnosis was written in 80% of operative notes pre-implementation of RCS guidelines; whereas, 100% of operative notes included operative diagnosis post-implementation. Pre-implementation of RCS guidelines, 78% of operative notes included the names of the anesthetist and the operative staff, 88% included assistant names, and 90% included the name of the operating surgeon; whereas, post-implementation of guidelines, these numbers increased to 92%, 93%, and 99%, respectively. Before implementation of RCS guidelines, only 6% of operative notes mentioned the operation type (e.g., emergency/elective), 88% mentioned the date of the operation, and 62% mentioned the time of the operation; whereas, post-implementation of guidelines, 100% of the operative notes mentioned the operation type, date, and time (Figure 3).

Pre-implementation of RCS guidelines, 10% of operative notes mentioned complications as compared to 94% post-implementation. Pre-implementation of guidelines, 7% of operative notes mentioned any extra procedure, and 2% mentioned if any tissue was removed or altered during surgery; whereas, post-implementation, 85% mentioned any extra procedure, and 70% mentioned tissue being removed or altered. Pre-implementation, 70% of operative notes mentioned the use of a prosthesis, 12% included details of closure techniques; whereas, post-implementation, 98% mentioned prosthesis use, and 93% mentioned closure technique details. Post-implementation of RCS guidelines, 90% of operative notes included details on whether deep venous thrombosis (DVT) prophylaxis was given to patients or not; whereas, pre-implementation, there was no mention of DVT prophylaxis. Similarly, there was no mention of time out in operative notes before the implementation of RCS guidelines; post-implementation, 100% of operative notes had time out in them. Before the implementation of RCS guidelines, 80% of operative notes included detailed postoperative orders, and 90% included signatures; whereas, post-implementation, 100% included postoperative orders and 95% included signatures (Table 1). Assuming a null hypothesis, the P-value was found to be 0.001 < P < 0.005, demonstrating a statistical significance in the improvement of data point inclusion between the pre- and post-audited groups of data.

## Discussion

Notation is part and parcel of medicine [5]. Well-written notes carry great importance in patient care, as they directly impact the follow-up care. The sole account of events occurring during surgery are operative notes [6]. Documentation is pivotal in surgery as circumstances may vary every second. [5]. Handwritten notes of operations may appear effortless; however, in actuality, writing notes requires dedication and is a challenging task. Notes written after surgery are necessary for post-operative care. These notes also provide a prerequisite for audit, inquisition study, and medico-legal intentions [5].

In this study, 500 operative notes were studied before and after the implementation of RCS guidelines. The operative notes improved many-fold after the implementation of RCS guidelines. Every minor detail of surgery was documented, as there was a separate column for each (two from RCS guidelines). An improved notation was observed when certain headings were combined in operative notes. Our observations correlated with the observations of Abbas et al., and for appendectomies conducted laparoscopically, certain proformas were used [7]. This displayed meaningful improvidence in notes.

Before the implementation of RCS guidelines, 72% of operative notes included pre-operative findings, and 80% included operative diagnosis, which improved after implementation of RCS guidelines. This was in accordance with the study by Sweed et al., who described in their study that proforma operative notes are more beneficial for proper documentation and showed that proper post-surgical orders on patient files reduced confusion among health care professionals in postoperative management [8]. Our audit improved postoperative order notation to 100%.

Documentation has a paramount role in litigation in practical medicine [9]. There is an increasing trend for doctors who have been forced to practice defensive medicine [10]. Easy interpretation and thorough records are vital inpatient care and can reduce the need for medical professionals to narrate the necessary information on the treatment provided. This portrayal of thorough documentation helps hospitals work more efficiently as time and resources are not wasted by seeking information about patients' treatment.

A limitation of our audit was the lack of computer-generated notes, although illegibility was not included in our audit. Studies have shown that computer-generated notes reduce legibility when compared to handwritten notes [11]. However, computer-generated notes require more resources, such as a proper up-to-date computer with the necessary software. Regardless of these factors, Coughlan et al. demonstrated that computer-generated notes are superior to handwritten notes [12]. Handwritten notes also consumed more time when they were formatted to RCS guidelines as more information needed to be noted. Despite these limitations, the quality of operative notes improved drastically in District Headquarter Hospital in Rawalpindi, Pakistan. It elucidated a closed-loop audit, with areas of improved and better notation.

## Conclusions

Well-structured and comprehensive proforma-based operative details are extremely beneficial. Marked improvement was seen in areas such as duration and post-operative orders. The greatest improvement was seen in major areas such as DVT prophylaxis and time out. Although only handwritten notes were collected, a computer-generated approach will further reduce the illegibility of these documents.
